# Erk Inhibition as a Promising Therapeutic Strategy for High IL-8-Secreting and Low SPTAN1-Expressing Colorectal Cancer

**DOI:** 10.3390/ijms25115658

**Published:** 2024-05-23

**Authors:** Clara Meier, Gianluca La Rocca, Virginia Nawrot, Beate Fißlthaler, Sarah J. Overby, Kai Hourfar, Guido Plotz, Christian Seidl, Paul Ziegler, Peter Wild, Stefan Zeuzem, Jürgen Brieger, Elke Jäger, Achim Battmann, Angela Brieger

**Affiliations:** 1 Biomedical Research Laboratory, Medical Clinic 1, University Hospital, Goethe University Frankfurt, 60590 Frankfurt, Germany; clmeier@med.uni-frankfurt.de (C.M.); gianlucalarocca2000@gmail.com (G.L.R.); virginia@med.uni-frankfurt.de (V.N.); overby@med.uni-frankfurt.de (S.J.O.); plotz@med.uni-frankfurt.de (G.P.); zeuzem@em.uni-frankfurt.de (S.Z.); 2 Centre for Molecular Medicine, Institute for Vascular Signalling, Goethe University Frankfurt, 60590 Frankfurt, Germany; fisslthaler@em.uni-frankfurt.de; 3 German Red Cross Blood Service Baden-Württemberg-Hessen, Institute for Transfusion Medicine and Immunohematology, Goethe University Frankfurt, 60590 Frankfurt, Germany; k.hourfar@blutspende.de (K.H.); c.seidl@blutspende.de (C.S.); 4 Dr. Senckenberg Institute of Pathology, University Hospital, Goethe University Frankfurt, 60590 Frankfurt, Germany; ziegler@med.uni-frankfurt.de (P.Z.); peter.wild@ukffm.de (P.W.); 5 Department of Otorhinolaryngology, University Medical Center Mainz, 55131 Mainz, Germany; brieger@uni-mainz.de; 6 Department of Oncology and Hematology, Hospital Nordwest, 60488 Frankfurt, Germany; jaeger.elke@khnw.de; 7 Department of Pathology, Hospital Nordwest, 60488 Frankfurt, Germany; batcave66@gmx.de

**Keywords:** colorectal cancer, non-erythrocytic spectrin alpha II, interleukin-8, epithelial–mesenchymal transition, tumor microenvironment

## Abstract

Tumor recurrence and drug resistance are responsible for poor prognosis in colorectal cancer (CRC). DNA mismatch repair (MMR) deficiency or elevated interleukin-8 (IL-8) levels are characteristics of CRCs, which have been independently correlated with treatment resistance to common therapies. We recently demonstrated significantly impaired therapeutical response and increased IL-8 release of CRC cell lines with reduced expression of MMR protein MLH1 as well as cytoskeletal non-erythrocytic spectrin alpha II (SPTAN1). In the present study, decreased intratumoral MLH1 and SPTAN1 expression in CRCs could be significantly correlated with enhanced serum IL-8. Furthermore, using stably reduced SPTAN1-expressing SW480, SW620 or HT-29 cell lines, the RAS*-*mediated RAF*/*MEK*/*ERK pathway was analyzed. Here, a close connection between low SPTAN1 expression, increased IL-8 secretion, enhanced extracellular-signal-regulated kinase (ERK) phosphorylation and a mesenchymal phenotype were detected. The inhibition of ERK by U0126 led to a significant reduction in IL-8 secretion, and the combination therapy of U0126 with FOLFOX optimizes the response of corresponding cancer cell lines. Therefore, we hypothesize that the combination therapy of FOLFOX and U0126 may have great potential to improve drug efficacy on this subgroup of CRCs, showing decreased MLH1 and SPTAN1 accompanied with high serum IL-8 in affected patients.

## 1. Introduction

Cancer is still one of the most common causes of death worldwide, and the resistance of tumors to standard chemotherapies is a major problem. The C-X-C motif chemokine ligand 8 (interleukin-8, IL-8) has been identified as the most extensively and significantly upregulated chemokine in colorectal cancer (CRC), and seems to be associated with drug resistance [1,2]. IL-8 contributes to CRC growth, invasion, metastasis and angiogenesis as well as neutrophilic tumor invasion [3,4]. The underlying molecular mechanisms, however, remain insufficiently understood. It is known that IL-8 plays an important role in the tumor microenvironment (TME) [5]. The composition of the TME strongly contributes to the regulation of the tumor epithelial–mesenchymal transition (EMT), a modification process of epithelial cells accompanied with loss of cell polarity and adhesion. In the TME, IL-8 is synthesized by different cell types, such as infiltrating immune, stromal, as well as tumor cells [6,7]. IL-8 can exert both autocrine and paracrine functions in TMEs. Autocrine IL-8 was demonstrated to induce the EMT by the IL-8–IL-8 receptor axis. A positive autocrine IL-8 loop has been shown to maintain the mesenchymal behavior of tumor cells that already passed the EMT. This maintenance of mesenchymal behavior is mainly accomplished by the serine/threonine protein kinase B (PKB, AKT) [8], the mitogen-activated protein kinase/extracellular-signal-regulated kinase (MAPK/ERK) [9] and the Janus-kinase 2/signal transducer and activator of transcription 3 (JAK2/STAT3) signaling pathways [10]. Paracrine IL-8, secreted by macrophages, has been detected to induce EMT in hepatocellular carcinoma cells via the JAK2/STAT3 pathway and the upregulation of N-Cadherin and zinc finger protein snail [10]. The EMT process, in turn, is accompanied with a massive modulation of the cytoskeleton by dynamic changes in cytoskeletal components like microfilaments, intermediate filaments and microtubules in affected tumor cells. High amounts of IL-8 are strongly correlated with EMT, which in parallel are associated with significant changes in the cytoskeleton of cells during tumor progression [11]. 

From a therapeutic point of view, it is important to consider that high IL-8 concentrations seem to be associated with resistance to cancer immunotherapies [12]. IL-8 has been assigned a role of promoting a favorable TME for tumor immune evasion by immune suppression. Furthermore, it is associated to induce a NET formation, which might envelope tumor cells and prevent contact with *CD8^+^* cytotoxic T *cells* and Natural killer cells [13]. Immunotherapy, more precisely the application of immune checkpoint inhibitors (ICIs), is used for CRCs with certain characteristics in order to reactivate the immune system to kill tumor cells. In addition to others, the US Food and Drug Administration (FDA) approved immunotherapy for the treatment of metastasized DNA mismatch repair (MMR)-deficient CRCs in 2017 [14]. MMR deficiency is due to germline mutations in MMR genes (most often *MutL homolog 1* (*MLH1*) or *MutS homolog 2* (*MSH2*)) [15] or of sporadic origin, and is caused by hypermethylation of the *MLH1* gene promoter, associated with a V600E missense mutation in the *BRAF* oncogene [16]. It is noteworthy that less than 50% of patients with MMR-deficient CRCs show a positive response to immunotherapy. The reason for this has not yet been clarified [17]. 

Recently, we showed that MLH1-deficient CRCs frequently harbor reduced expression levels of the important cytoskeletal protein non-erythrocytic spectrin alpha II (SPTAN1) [18]. Additionally, CRC cell lines, in which reduced MLH1 and reduced SPTAN1 expression was induced by stable transduction of a SPTAN1-specific shRNA, express and secrete significantly increased IL-8 levels. High IL-8 levels were shown to induce enhanced migration of neutrophilic granulocytes [19] and impaired response to common chemotherapy [20]. 

In the present study, we determined IL-8 values in the sera of patients with CRC before surgery and correlated these levels with the amounts of MLH1, SPTAN1 and Programmed Cell Death Ligand 1 (PD-L1) in corresponding tumors after resection. Furthermore, we analyzed the impact of decreased SPTAN1 expression on key molecular processes of tumor progression by examining the Rat sarcoma (RAS)*-*mediated rapidly accelerated fibrosarcoma/mitogen-activated protein kinase kinase/extracellular-signal-regulated kinase (RAF*/*MEK*/*ERK) pathway, which has emerged as a potential inducer of EMT. We propose new individualized therapeutic approaches that have the potential to overcome drug resistance in tumors with these genetic characteristics.

## 2. Results

### 2.1. Reduced MLH1 as Well as Reduced SPTAN1 Expression in CRCs Are Significantly Correlated with Enhanced IL-8 Levels in Sera of Corresponding Patients

**Figure 1:**
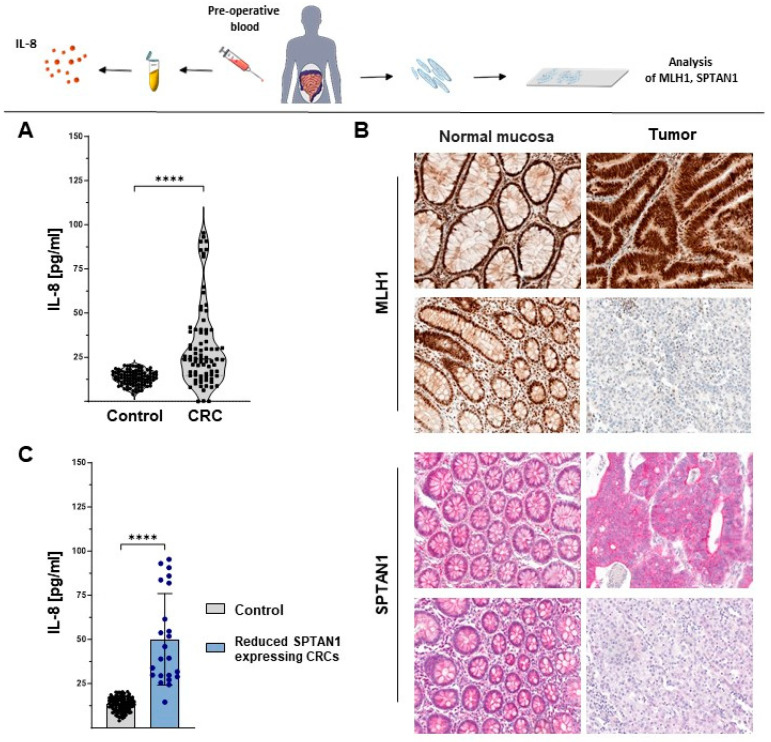
Reduced SPTAN1 expression correlates with enhanced serum IL-8 concentrations in CRCs. Sera of 80 colorectal cancer (CRC) patients were collected before tumor resection and compared with 100 control sera. (**A**) Interleukin-8 (IL-8) levels were determined by Enzyme-Linked Immunosorbent Assay (ELISA) measurements. After surgery, paraffin-embedded CRCs (n = 75) of corresponding patients were analyzed for MutL homolog 1 *(*MLH1) and non-erythrocytic spectrin alpha II (SPTAN1) expression via immunohistochemical staining. (**B**) Exemplary images are shown of tumor surrounding normal mucosa (left side) and corresponding tumor (right side) of MLH1 (upper part) and SPTAN1 (lower part) stained on the same slide (20-fold magnification). For both proteins, a proficient (upper images) and a deficient/reduced (lower images) expressing tumor are shown, respectively. (**C**) IL-8 levels of reduced SPTAN1-expressing CRCs are significantly increased in comparison to the controls. *p*-values were calculated via Mann–Whitney U test. **** *p* < 0.0001.

We previously demonstrated a close correlation between MLH1 deficiency, low SPTAN1 expression, tumor progression and the degree of metastasis [18]. Recently, we were able to show a strong connection between reduced SPTAN1 expression and elevated IL-8 secretion in vitro [19] and postulated IL-8 as a promising marker protein and potential target for personalized treatment optimization. In the current study, the correlation of MLH1, SPTAN1 and IL-8 was examined in vivo. For this purpose, IL-8 levels of the sera of 80 CRC patients collected prior to tumor resection were analyzed and compared to IL-8 levels of the sera of healthy controls (n = 100) by Enzyme-Linked Immunosorbent Assay (ELISA) measurements. A significant increase in serum IL-8 could be detected in patients compared to the controls (Figure 1A; *p* < 0.0001). In contrast, the levels of IL-1β, IL-6 and TNF-α were in the normal range of clinical diagnostics and were not elevated (Figure S1). To investigate the correlation of serum IL-8 to MLH1 and SPTAN1 expression of the corresponding CRCs, MLH1 as well as SPTAN1 expression were determined via immunohistochemical staining (n = 75; immunohistochemical staining was not possible in five tumors) (example shown in Figure 1B), and quantified using a simple computer-based algorithm in ImageJ version 1.54f (see Section 4). In line with previously published data [18], we verified a significant correlation between SPTAN1 and MLH1 expression in CRCs (*p* = 0.0038). Furthermore, a significant correlation was observed between reduced MLH1 (*p* < 0.0001) as well as reduced SPTAN1 (*p* = 0.0002) expression levels with increased IL-8 concentrations in CRC patients. IL-8 levels of the sera of patients with reduced SPTAN1-expressing CRCs were significantly increased in comparison to the controls (Figure 1C, *p* < 0.0001). Similar effects were detected for MLH1 (Figure S1).

### 2.2. Enhanced PD-L1 Expression Is Not Associated with Reduced Expression of MLH1 and SPTAN1 or Increased IL-8 Levels in CRCs 

**Figure 2:**
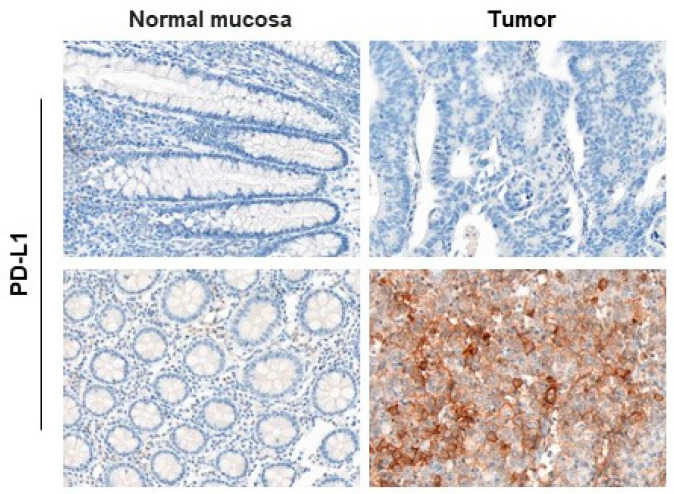
Enhanced PD-L1 expression in CRCs does not correlate with enhanced serum IL-8 or reduced MLH1 or SPTAN1 expression in corresponding patients. After surgery, paraffin-embedded CRCs (n = 75) were analyzed for Programmed Cell Death Ligand 1 (PD-L1) expression via immunohistochemical staining. Exemplary images are presented of tumor surrounding normal mucosa (left side) and corresponding tumor (right side) of PD-L1 stained on the same slide (20-fold magnification). A PD-L1-deficient (upper image) and an enhanced PD-L1-expressing (lower image) tumor are shown. Correlation analysis revealed no significant association between PD-L1 expression and IL-8 serum levels nor with MLH1 and SPTAN1 expression of the corresponding CRCs.

To uncover a potential connection between IL-8, MLH1, SPTAN1 and PD-L1 in our cohort, we determined PD-L1 by immunohistochemical staining (Figure 2). PD-L1 expression intensity was correlated for each patient to MLH1 and SPTAN1 intensity. In addition, PD-L1 tumor levels of every patient were correlated to IL-8 serum levels. We detected no correlation between high PD-L1 expression and reduced MLH1 (*p* = 0.33) or SPTAN1 expression (*p* = 0.14). Additionally, we could not correlate high IL-8 serum concentrations with enhanced PD-L1 expression in CRC patients (*p* = 0.075).

### 2.3. Reduced SPTAN1 Expression Is Associated with Enhanced ERK Phosphorylation and Increased IL-8 Secretion In Vitro

**Figure 3:**
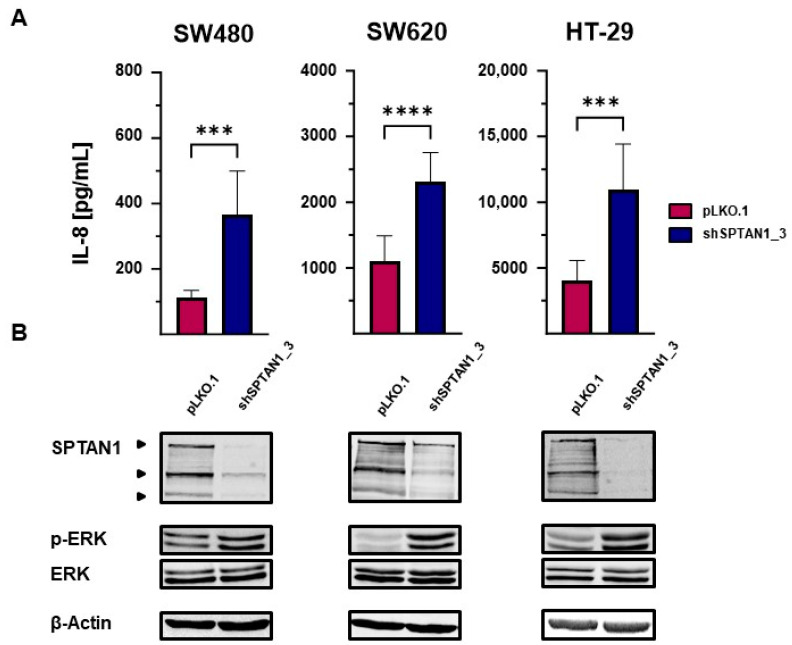
Significantly reduced SPTAN1 expression is associated with significantly increased ERK activation. Stable transduction of shRNA was used to reduce SPTAN1 expression in SW480, SW620 and HT-29 colon cancer cells; stable transduction of non-mammalian shRNA (pLKO.1) served as control. These reduced SPTAN1-expressing SW480, SW620 and HT-29 and corresponding control cell lines were cultivated and harvested. (**A**) The IL-8 secretion was investigated using ELISA measurements (n = 9) and (**B**) the expressions of SPTAN1, extracellular signal-regulated kinase (ERK) and the activated, phosphorylated form of ERK (p-ERK) were analyzed via Western blotting (n ≥ 3). As shown, full length SPTAN1 is recognized as a protein of 284 kD, cleavage products of SPTAN1 run at 150 kD and 120 kD. The IL-8 secretions of reduced SPTAN1-expressing cells (SW480 shSPTAN1_3; SW620 shSPTAN1_3; HT-29 shSPTAN1_3) were significantly increased in comparison with corresponding pLKO.1-transduced controls. Decreased SPTAN1 expression was associated with significantly elevated ERK activation. *p*-values were calculated using unpaired *t*-test. The data shown are means ± SD, and the following *p*-values were considered as statistically significant: *** *p* < 0.001, **** *p* < 0.0001.

Our next goal was to determine the underlying molecular mechanisms that are responsible for the SPTAN1-dependent enhanced IL-8 levels. Therefore, we used shSPTAN1-transduced SW480, SW620 and HT-29 cells and first verified that these cells expressed significantly reduced SPTAN1 levels in comparison with the corresponding pLKO.1-transduced control cells (for all *p* < 0.0001; Figure S1B). In accordance with previously published data [19], the reduced SPTAN1-expressing cell lines (SW480, SW620 and HT-29, Figure 3B) showed significantly increased IL-8 levels compared to control cells (Figure 3A). To identify treatable changes that may be responsible for these enhanced IL-8 secretions, the activation of MAPK (ERK, p38) and the immunoglobulin kappa light-chain of activated B cells (NFκB) was determined in vitro via Western blotting (Figure 3B and Figure S2). The activated, phosphorylated form of ERK (p-ERK) was significantly enhanced in reduced SPTAN1-expressing SW480 (*p* = 0.0064), SW620 (*p* = 0.0487) as well as HT-29 (*p* = 0.0265) cell lines compared to the controls (Figure 3 and Figure S1B), while the expression of ERK was unchanged. In addition, there were no changes in phosphorylation of NFκB or p38 (Figure S1A). 

### 2.4. Significant Reduction in IL-8 Secretion and Cell Viability Can Be Induced by Inhibition of ERK in Decreased SPTAN1-Expressing CRC Cell Lines

**Figure 4:**
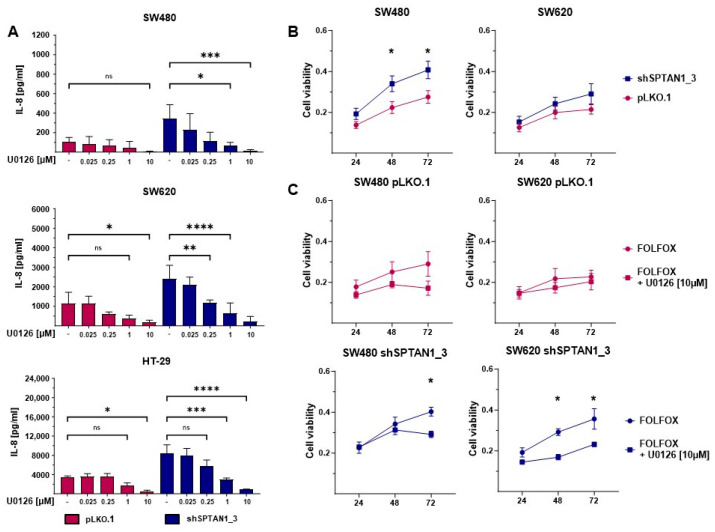
Inhibition of ERK significantly reduces IL-8 secretion and cell viability of decreased SPTAN1-expressing cells. 5 × 10^5^ of differential SPTAN1-expressing SW480, SW620 and HT-29 cells were incubated for 48 h, then treated with the indicated concentrations of the ERK specific inhibitor U0126 for 18h, followed by (**A**) ELISA measurements. Columns show IL-8 concentrations of reduced SPTAN1-expressing cell lines (SW480 shSPTAN1_3; SW620 shSPTAN1_3; HT-29 shSPTAN1_3) (blue columns) in comparison to the pLKO.1-transduced controls (pink columns). The inhibition of ERK by U0126 treatment significantly reduced IL-8 secretion, most efficiently at a concentration of 10 µM. (**B**) The treatment efficacy on cell viability was assessed using 3-(4,5-dimethylthiazol-2-yl)-2,5-diphenyl-2H-tetrazolium bromide (MTT). Cells were seeded 1 × 10^4^ per well in 96-well culture plates. After cell adhesion, cells were preincubated with or without 10 µM U0126 for 18 h. Subsequently, the culture medium was removed and the cells were treated with Folinic acid, fluorouracil and oxaliplatin (FOLFOX) with or without supplementation of 10 µM U0126 for 24 h, 48 h and 72 h. Reduced SPTAN1-expressing cells showed a significantly weaker response than corresponding control cells to FOLFOX treatment. Graphs indicate the results (mean ± SEM) of n = 9 (SW480) and n = 5 (SW620). (**C**) The reduced response of the decreased SPTAN1-expressing cells was significantly improved by the combination treatment of U0126 and FOLFOX; n = 3. *p*-values were calculated using one-way ANOVA. The following *p*-values were considered as statistically significant: ns = not significant, * *p* < 0.05, ** *p* < 0.01, *** *p* < 0.001, **** *p* < 0.0001.

In order to identify therapeutic options to reduce IL-8 secretion, the treatment responses of reduced SPTAN1-expressing SW480, SW620 as well as HT-29 cell lines on different inhibitors were analyzed. To investigate the signaling pathways responsible for the increased IL-8 concentrations, the cells were respectively stimulated with specific inhibitors for ERK (U0126), p38 (SB202190), Jun N-terminal kinase (JNK) (SP600125) and NFκB (Dexamethasone) with the indicated concentrations for 18 h. Subsequently, cell supernatants were collected and IL-8 concentrations were analyzed using ELISA (Figure 4A and Figure S3). 

As already demonstrated (Figure 3A), IL-8 secretion was significantly higher in all reduced SPTAN1-expressing cell lines (Figure 4A, blue columns) compared to the controls (Figure 4A, pink columns). The inhibition of ERK by U0126 led to a dose-dependent and significant reduction in IL-8 secretion that was most efficient in the reduced SPTAN1-expressing cell lines. Treatment with 10 µM U0126 was able to almost completely reduce IL-8 secretion in all cell lines (Figure 4A). In contrast, no change in IL-8 secretion could be detected after inhibition of JNK and NFκB (Figure S3), and only reduced SPTAN1-expressing HT-29 cells showed a response on p38 inhibition (Figure S3). Using an MTT assay, we demonstrated that reduced SPTAN1-expressing SW480 and SW620 cells responded poorly to Folinic acid, fluorouracil and oxaliplatin (FOLFOX) treatment compared to the control cells (Figure 4B). These findings were consistent with clinical data and previously published data on HT-29 cells [20]. Hence, we investigated whether the increased ERK activation in these cells plays a role for their poor FOLFOX response. Therefore, cells were preincubated with or without 10 µM U0126 and subsequently treated with FOLFOX supplemented with or without 10 µM U0126 for 24 h, 48 h or 72 h. Cell viability was assessed via MTT. The combination of FOLFOX and U0126 significantly reduced the cell viability of the reduced SPTAN1-expressing cells (blue lines) but not of the control cells (pink lines) (Figure 4C). After this combined treatment, both cell lines (reduced SPTAN1-expressing and control cells) were at a comparable level. 

### 2.5. Combination Treatment of U0126 and FOLFOX Led to Significantly Decreased Long-Term Survival of CRC Cell Lines

**Figure 5:**
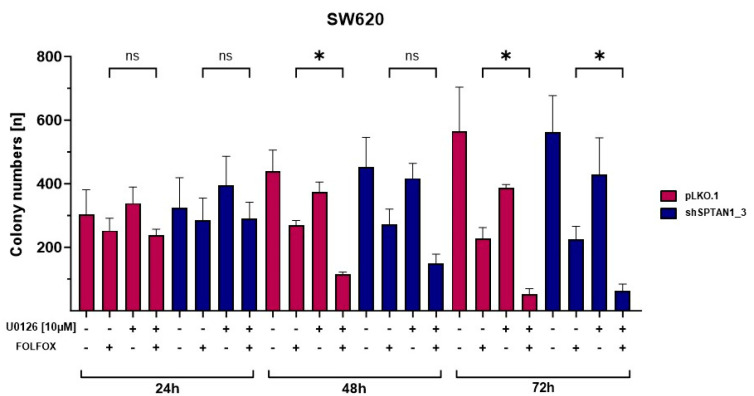
FOLFOX and U0126 co-treatment significantly reduces long-term survival of reduced SPTAN1-expressing cells. Cells seeded at 1 × 10^3^ per cell culture flask (surface area 25 cm^2^) were incubated until adhesion and cultivated in medium supplemented with or without 10 μM of U0126 for 18 h. Then, cells were treated with FOLFOX supplemented with or without 10 μM of U0126 for 24 h, 48 h and 72 h. Subsequently, the medium was replaced and cells were cultivated in medium without supplements for a further seven days. Colonies were fixed with 4% paraformaldehyde and stained with 0.5% crystal violet. The number of colonies was quantified using the COLCOUNT^TM^ system and Oxford Optronix ColCount software version 4.3.5.1. Columns show the mean number of colonies ± SD of reduced SPTAN1-expressing cells (shSPTAN1_3 blue columns) in comparison to the pLKO.1-transduced controls (pink columns). Co-treatment of U0126 and FOLFOX for 48 h as well as 72 h significantly inhibited the colony formation of all cell lines. *p*-values were calculated using one-way ANOVA; n = 3. The following *p*-values were considered as statistically significant: ns = not significant, * *p* < 0.05.

A decreased sensitivity and therapy resistance to FOLFOX treatment has been shown in reduced MLH1- as well as reduced SPTAN1-expressing cells [20]. Therefore, we next examined the efficacy of treatment with U0126 on the long-term survival of cells expressing reduced levels of SPTAN1. Again, we preincubated reduced SPTAN1-expressing SW620 and SW480 cells and corresponding control cell lines with and without 10 µM U0126 for 18 h, and subsequently treated them with FOLFOX supplemented with or without 10 µM U0126 for 24 h, 48 h or 72 h. Afterwards, the cells were cultivated for one week in medium without supplements. The formation of colonies was assessed by colorimetric measurements. The combination therapy with U0126 and FOLFOX led to significantly reduced colony formation in both SPTAN1-reduced-expressing and control cells after 48 as well as 72 h of treatment compared to standard FOLFOX therapy (Figure 5). A similar trend could be observed in differential expressing SW480 cell lines (Figure S4). 

### 2.6. CRC Cell Lines with Reduced SPTAN1 Expression Exhibit Significantly Lower Cell Migration and a Mesenchymal Phenotype

**Figure 6:**
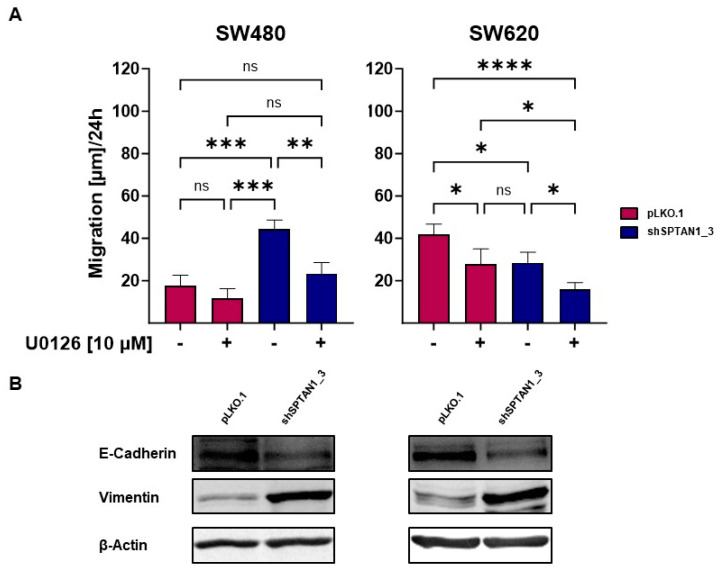
Inhibition of ERK impairs cell migration and reduced SPTAN1 expression is associated with a mesenchymal phenotype. A wound migration assay was used to test the efficacy of ERK inhibition by 10 μM U0126 treatment on cell mobility of differential SPTAN1-expressing cells. The migratory rates of reduced SPTAN1-expressing cells (SW480 shSPTAN1_3 and SW620 shSPTAN1_3), as well as corresponding pLKO.1-transduced control cells, were compared by measuring the alteration of the wound width at different time points (0 h and 24 h). (**A**) Graphs show the migration ability of differential expressing SW480 and SW620 cells after 24 h. U0126 was able to significantly reduce the mobility of reduced SPTAN1-expressing SW480 as well as SW620 cells. (**B**) To investigate EMT, differential SPTAN1-expressing SW480 and SW620 cell lines were analyzed via Western blotting for E-Cadherin and Vimentin expression. The data shown are means ± SD; the following *p*-values were considered as statistically significant: ns = not significant, * *p* < 0.05, ** *p* < 0.01, *** *p* < 0.001, **** *p* < 0.0001. n ≥ 3.

To assess the short time effect of ERK inhibition on cell mobility, migration of differential SPTAN1-expressing cells was tested via scratch wound migration assay. After application of the scratches, the cells were incubated in medium supplemented with or without 10 µM U0126. The migration of the cells was examined after 24 h. Without treatment, the mobility of reduced SPTAN1-expressing SW480 cells was significantly increased in comparison to the controls, but treatment with U0126 significantly reduced the migration ability of reduced SPTAN1-expressing SW480 cells close to the levels of the control cell line (Figure 6A, left graph; Figure S5, lower panels). In contrast, untreated SW620 control cells showed significantly more cell mobility than untreated reduced SPTAN1-expressing SW620 cells. However, treatment with U0126 led to a significant mobility reduction in both SPTAN1-reduced-expressing and control SW620 cells. The migration of reduced SPTAN1-expressing SW620 was also significantly reduced in comparison to the high migration ability of treated control cells (Figure 6A, right panel; Figure S5, upper panels). 

The EMT is a process that allows polarized epithelial cells to undergo multiple changes associated with loss of epithelial differentiation and the gain of a mesenchymal phenotype [21]. The expression of many genes has been shown to be associated with EMT to induce cell migration and metastasis. In order to identify if SPTAN1 reduction is associated with a mesenchymal phenotype, we determined the expression levels of E-Cadherin, a very well-known marker of EMT changes in epithelial cells [22], and Vimentin, a type III intermediate filament protein, which is often upregulated in mesenchymal cells and has been pathologically associated with tumor invasion and metastasis [23]. As shown in Figure 6B, E-Cadherin was downregulated in reduced SPTAN1-expressing SW480 as well as SW620 cells, while Vimentin expression was upregulated in cell lines with decreased SPTAN1 levels. 

## 3. Discussion

Despite significantly improved treatment options due to the availability of ICIs, therapy resistance is still a major problem for the treatment of CRCs (reviewed by [24]); although MMR deficiency in CRCs has been established as a striking biomarker for response to ICIs, MMR-deficient CRCs frequently present primary resistance to ICIs and early tumor progression [25,26,27,28]. The reasons for non-response are still unclear. One frequently used immunotherapeutic target to predict a response of MMR-deficient CRCs to ICIs is the amount of PD-L1 expression. Increased expression of PD-L1 in the early stage of immunotherapeutic treatment seems to serve as a positive biomarker for PD-L1 blockade efficacy [29]. In our patients’ cohort, we did not detect a significant correlation between reduced MLH1 expression (or SPTAN1) and increased PD-L1. Although we do not have clinical data on our cohort’s response to ICIs, one may assume that our patients would not benefit from this type of therapy.

In addition to low tumor PD-L1 expression, high systemic and tumor-associated IL-8 levels have been demonstrated to correlate with reduced clinical benefit to treatment with ICIs [29]. This effect has been shown, for example, in metastatic renal cell carcinoma [30] and advanced melanoma or non small-cell lung cancer [31]. Since IL-8 is able to inhibit not only apoptosis-inducing proteins caspase 3 and 9, but also PARP, and promotes the expression of Bcl-2, Survivin or Bcl-xl, -xs [32,33,34]; tumor-associated enhanced IL-8 may directly counteract apoptosis and ICI therapy response. In addition to this, elevated serum IL-8 levels have been associated with increased circulating and tumor-associated NET formation in CRCs [35]. In the described context, our group previously made two important observations. Firstly, we identified a close correlation between MLH1 deficiency and low SPTAN1 expression in CRCs [18]. Secondly, we detected significantly enhanced IL-8 secretion in CRC cell lines harboring MLH1 and SPTAN1 reduction in vitro, which was correlated with impaired response to common chemotherapy [19]. In the present study, we were again able to define a group of CRCs that showed significant correlation between MLH1 and SPTAN1. Most interestingly, we could moreover verify in vivo that decreased MLH1 as well as SPTAN1 expression was accompanied with significantly enhanced IL-8 levels in corresponding patients’ sera, while other interleukins like IL-1β, IL-6 and TNF-α remained unchanged. 

Therefore, we speculated that increased IL-8 secretion might serve as a promising marker protein to encircle ICI therapy-resistant CRCs, and may be a starting point to develop a new therapy. To identify IL-8-associated new potential therapeutic targets, we next focused on the underlying in vitro molecular mechanism leading to SPTAN1-dependent enhanced IL-8 expression and secretion using different CRC cell line models that mimick the described special CRC phenotype. Thus, different signaling pathways that are associated with IL-8 expression and secretion (MAPK, NFκB) were analyzed and—as the only one—significantly increased ERK activation was clearly and consistently identified in different, low SPTAN1-expressing CRC cell models accompanied with enhanced IL-8 secretion. 

In addition, an advanced mesenchymal phenotype in association to low SPTAN1 expression could be manifested. These observations are in line with previously published data demonstrating that high amounts of IL-8 are strongly correlated with EMT (reviewed in [36]). Furthermore, the increase in IL-8 and an advanced EMT stage have been demonstrated to be associated with significant changes in the cytoskeleton of cells during tumor progression [11]. Since SPTAN1 is an important cytoskeletal protein that plays a major role in cell polarity, a direct correlation between increased IL-8 expression and reduced SPTAN1 expression could be assumed in our tumor model. 

Several clinical trials are focusing on ERK inhibition to enhance therapy, particularly in drug-resistant tumors such as gastric cancer and hepatocellular carcinoma [37,38]. Based on our data showing that increased ERK activation is associated with reduced SPTAN1 expression, we next selected ERK inhibition as a target for optimizing therapy in CRC cells expressing reduced SPTAN1. Therefore, the combinational therapy of FOLFOX and ERK inhibition was not only able to significantly inhibit cell viability and colony formation, but was also capable of reducing cell migration in our CRC cell line models mimicking the described special CRC phenotype. Interestingly, IL-8-mediated induction of NET formation in neutrophils is regulated by ERK [39,40]. Thus, the reduction in IL-8 via ERK inhibition represents a potential way to reduce the tumor progression of SPTAN1-reduced-expressing tumors at different levels.

## 4. Materials and Methods

### 4.1. Patients

Patient samples (n = 80) were obtained from the Hospital Nordwest, 60488 Frankfurt, Germany. The sera of patients were isolated from blood samples and collected before surgery and prior exposure to neoadjuvant chemoradiotherapy. Within 4 h, samples were frozen at −80 °C and stored until use for different ELISAs. In addition, paraffin-embedded tissue of resected, well-characterized corresponding colorectal tumors of the same patient cohort, as well as adjacent normal colonic mucosa tissue, were used for immunohistochemical analysis. As a control for serum analysis, serum was isolated from blood samples of commercially available anonymous healthy donors (n = 100) obtained from the German Red Cross Blood Donor Service Baden–Wuerttemberg–Hessen, Institute of Transfusion Medicine and Immunohematology, Goethe University Frankfurt, 60590 Frankfurt, Germany, and frozen at −80 °C within 4 h after collection and stored until use. 

The study was approved by the local ethics committee of University Hospital Frankfurt (business no.: 4/09, project number SGI-1-2018), and all patients and healthy donors provided written informed consent.

Basic clinical characteristics such as gender, age at diagnosis, IL-8 serum concentrations before tumor resection, as well as MLH1, SPTAN1 and PD-L1 expression differences between tumor and tumor surrounding normal mucosa are listed in Table S1.

### 4.2. Cell Lines

In this study, stably reduced SPTAN1-expressing SW480, SW620 and HT-29 cells as well as corresponding control vector-transduced cell lines were used, which have already been published [19]. The cell lines were generated by lentiviral transduction according to the MISSION Lentiviral Packaging Mix Technical Bulletin. Therefore, SW480 (ATCC #CCL-228), SW620 (ATCC #CCL-227) and HT-29 (ATCC #HTB-38) cells were plated at a density of 1 × 10^5^ cells per well and transduced with 3 μg of short hairpin RNA (shRNA) targeting SPTAN1 (MISSION^®^ shRNA TRCN0000053669), delivered through a viral vector. As a control, SW480, SW620 and HT-29 cells were transduced with the same amount of viral vector containing non-mammalian shRNA (MISSION pLKO.1-puro vector SHC002V). Transduced cells were selected for puromycin-containing (5 μg/mL) cell culture medium.

Afterwards, all cell lines were grown in DMEM (Dulbecco’s Modified Eagle Medium, Gibco, Paisley, Renfrewshire, UK) with 10% FCS (Catalog number F0926, Sigma-Aldrich, Darmstadt, Hessen, Germany) and 1% penicillin–streptomycin (Sigma-Aldrich, USA). The cells were tested frequently for mycoplasma and characterized in December 2023 by short tandem repeat (STR) profiling, as indicated by the DSMZ online catalogue (https://www.dsmz.de/collection/catalogue/microorganisms/catalogue, accessed on 19 May 2024). STR profiling of the 8 STR loci was performed as described [41].

### 4.3. Inhibitor Studies and ELISA Measurements

The inhibition experiments were performed with different CRC cell lines (SW480, SW620 and HT-29), either with stable reduced SPTAN1 expression or transduced with pLKO.1 vector for control. Before treatment, 5 × 10^5^ cells per 6-well culture plates were seeded and incubated for 48 h at 37 °C in 5% CO_2_. Subsequently, media were replaced and cells were treated with the indicated concentrations of either U0126 (MEK1/2 inhibitor, LC Laboratories, Woburn, MA, USA), SB202190 (p38α/β MAPK inhibitor, Hölzel Diagnostika, Köln, Deutschland), SP600125 (SAP/JNK MAPK inhibitor, Hölzel Diagnostika, Köln, Deutschland) or dexamethasone (NFκB inhibitor, Adooq Bioscience, Irvine, CA, USA). After 18 h of incubation, the cell supernatants were collected followed by ELISA measurements using the BD OptEIA Human IL-8 ELISA Set (BD Bioscience, San Diego, CA, USA), Human IL-1β/IL-1F2 DuoSet^®^ ELISA, Human TNF-alpha DuoSet^®^ ELISA and Human IL-6 DuoSet^®^ ELISA (R&D Systems, Minneapolis, MN, USA) kits according to the manufacturers’ protocols. 

ELISA measurements of human serum samples were performed using the Human IL-8/CXCL8 Quantikine HS ELISA, Human TNF-alpha Quantikine HS ELISA, Human IL-1 beta/IL-1F2 Quantikine HS ELISA and Human IL-6 Quantikine HS ELISA (all from R&D Systems, Wiesbaden, Germany) kits according to the manufacturers’ recommendations. Duplicates of all samples were used for ELISA measurements as well as protein standard dilutions, together with negative controls. The optical density was detected using an EnVision 2104 Multilabel Plate Reader (PerkinELmer, Waltham, MA, USA). All of the experiments were performed at least 3 times.

### 4.4. Protein Extraction and Western Blotting

Denatured protein extracts of cells were prepared as previously described [42]. In brief, 5 × 10^5^ cells were seeded in 6-well cell culture plates and incubated for 48 h at 37 °C in 5% CO_2._ Afterwards, the cells were harvested and washed twice with PBS and centrifuged at 3000 g for 3 min at 4 °C. The cells were solved in lysis reagent (CelLytic^TM^ M Lysisbuffer, Sigma-Aldrich, St. Louis, MO, USA) combined with cOmplete^TM^ Protease Inhibitor Cocktail (Roche Pharma, Basel, Switzerland), incubated on ice for 5 min, lysed by ultrasound cavitation and a final centrifugation at 12,000× *g* for 10 min at 4 °C. The protein extracts were stored at −20 °C until use. Proteins (50 µg protein per lane) were separated on 10% polyacrylamide gels, followed by Western blotting on nitrocellulose membranes and antibody detection using standard procedures. Membranes were incubated with different primary antibodies (Table 1), followed by incubation with fluorescent-labeled secondary antibodies (anti-mouse IRDye^®^ 680 LT, anti-rabbit IRDye^®^ 680 LT, anti-mouse IRDye^®^ 800 CW, all from LI-COR Biosciences; Lincoln, NE, USA). All phosphorylated and unphosphorylated protein pairs were generated from the same experiment. All of the experiments were performed at least three times. For imaging, the FLA-9000 scanner (Fujifilm, Tokyo, Japan) was used. If indicated, Western blot band intensities were quantified using the image analysis software Multi Gauge version 3.2 (Fujifilm, Tokyo, Japan).

### 4.5. Determination of Cell Viability

The responses to different combination therapies were assessed using the 3-(4,5-dimethylthiazol-2-yl)-2,5-diphenyl-2H-tetrazolium bromide (MTT) colorimetric assay, as described previously [20]. Cells were seeded 1 × 10^4^ per well in 96-well culture plates (Greiner Bio-One, Frickenhausen, Deutschland). After cell adhesion, cells were preincubated with or without 10 µM U0126 for 18 h at 37 °C and 5% CO_2_. Subsequently, the culture medium was removed and the cells were treated with FOLFOX (40 µM fluorouracil, 0.008 µM oxaliplatin, 0.16 µM folic acid) with or without supplementation of 10 µM U0126 for 24 h, 48 h and 72 h. At the specified time points, culture media were replaced with media containing tetrazolium dye MTT (Sigma-Aldrich), at a final concentration of 833 µg/mL, and incubated for 2 h at 37 °C in 5% CO_2_. Afterwards, cells were treated with 100 µL of a decolorizing solution (DMSO, 0.6% acetic acid, 0.1 g/mL SDS). After 20 min, the absorbance was measured at 570 nm using an EnVision 2104 Multilabel Plate Reader (PerkinElmer, Waltham, MA, USA). Each experiment was repeated at least three times.

### 4.6. Colony-Forming Assay

To examine different treatment therapies and their impact on the long-term survival of treated cells, a colony formation assay was performed. Cells were seeded at 1 × 10^3^ cells per cell culture flask (surface area 25 cm^2^) and incubated until adhesion for 5 h at 37 °C and 5% CO_2_. Afterwards, cells were cultivated in medium supplemented with or without 10 μM of U0126 for 18 h at 37 °C in 5% CO_2_. Subsequently, cells were treated with FOLFOX (40 µM fluorouracil, 0.008 µM oxaliplatin, 0.16 µM folic acid) supplemented with or without 10 µM U0126 and incubated for 24 h, 48 h and 72 h. After these periods of time, the medium was replaced with culture medium without supplements, and the cells were incubated for a further seven days. Appropriate controls were included for each condition. Thereafter, the colonies were fixated with 4% paraformaldehyde and stained with 0.5% crystal violet. Finally, the cells were rinsed with PBS and dried at room temperature. The number of colonies was analyzed and quantified using the COLCOUNT^TM^ system (Oxford Optronix Ltd., Abingdon, UK) and Oxford Optronix ColCount software version 4.3.5.1. Each experiment was performed in duplicate and repeated at least three times.

### 4.7. Scratch Wound Migration Assay 

To analyze cell migration, the scratch wound migration assay and IncuCyte Zoom live cell imaging system (Essen Bioscience, Ann Arbor, MI, USA) were used. The seeding used was 6.5 × 10^4^ cells in 96-well ImageLock plates (Essen Bioscience, Ann Arbor, MI, USA), and the cells were grown to confluence under standard conditions. After 24 h, scratches were created simultaneously in all wells with a WoundMaker (Essen Bioscience, MI, USA) according to the manufacturer’s instructions. Immediately after the generation of the scratches, the medium was changed, and medium supplemented with or without 10 µM of ERK inhibitor U0126 was applied. The wound closures were scanned every 4 h and monitored over 24 h using Wound Width Analysis IncuCyte Software version 2020B, allowing for the exact identification of wound regions. The distance of cell migration in μm was calculated by the difference between the initial and the 24 h wound widths divided by 2. Each experiment was performed at least three times.

### 4.8. Immunohistochemical Staining

Expressions of different proteins were analyzed by immunohistochemical staining using paraffin-embedded, invasively growing colorectal tumor tissue, corresponding surrounding normal mucosa and external control tissue, according to standard procedures. In brief, 2 µm sections of representative samples were cut from paraffin-embedded sample blocks and collected onto X-tra^®^ microscope slides and oven-dried at 74 °C for 20 min. Subsequently, slides were stained for MLH1 using VENTANA^®^ anti-MLH1 (clone M1, Roche Diagnostics, Rotkreuz, Switzerland; dilution 1:50), for PD-L1 using anti-PD-L1 (clone CAL10, Zytomed Systems, Berlin, Germany; dilution 1:50) and for SPTAN1 using anti-spectrin αII (clone C-11, Santa Cruz Biotechnology, Heidelberg, Germany; dilution 1:100). Afterwards, the sections were incubated with a horseradish peroxidase-coupled secondary antibody, and then stained with chromogen 3,3-diaminobenzidine for MLH1 and PD-L1 or alkaline phosphatase for SPTAN1. Sections were counterstained using hematoxylin followed by 4 wash steps. The sections were covered using Tissue-Tek Film^TM^ (SAKURA, Umkirch, Deutschland). Immunohistochemical staining was examined using a Keyence microscope (Model BZ-9000; Keyence Co., Osaka, Japan).

### 4.9. Image Processing

Representative images of the immunohistochemical staining were obtained using a digital slide scanner (3DHISTECH, Sysmex, Budapest, Hungary). Subsequently, separate image sections of the tumor and tumor-surrounding normal colorectal tissue were created at a 20-fold magnification using the Case Viewer program (3DHISTECH, Sysmex, Budapest, Hungary). The semi-quantitative analysis of the images was carried out using ImageJ version 1.54f, as previously described [21]. In brief, the hematoxylin and eosin staining was separated from the staining of the target structure by color deconvolution. The images were converted into 8-bit greyscale images and inverted. Each pixel of the image was assigned an intensity value between 0 and 255. Finally, the mean intensity values for all pixels of an image were generated by measurements in ImageJ version 1.54f. For the subsequent calculation, the staining of the tumor was related to the surrounding normal tissue.

### 4.10. Statistical Analysis

All calculations were analyzed using the software GraphPad Prism 10 for Windows, version 7.04 (GraphPad Software, La Jolla, CA, USA). The correlation analyses were performed using Spearman rank correlation. Paired two-tailed *t*-tests followed by Welch correction for unequal variances or one-way ANOVA for normality and Mann–Whitney test for non-normally distributed data were used to assess statistical significance. The data are shown as mean ± SD, unless otherwise stated; the following *p*-values were considered statistically significant: * *p* < 0.05, ** *p* < 0.01, *** *p* < 0.001, **** *p* < 0.0001.

## 5. Conclusions

The in vivo data presented here show a significant correlation between reduced MLH1 as well as decreased SPTAN1 and significantly increased serum levels of IL-8 in the respective patients. Corresponding in vitro results demonstrate that the underlying molecular mechanism is due to an increased activation of ERK, which led to an advanced EMT. A combination of commonly used FOLFOX therapy and ERK inhibition by U0126 significantly improved the response of CRC cells in our model, and represents a promising new therapeutic option for the subgroup of MMR-deficient CRCs presented here, which may be able to escape ICI therapy. 

## Figures and Tables

**Table 1:** Used primary antibodies and target proteins.

Primary Antibody (Clone)	Target (Molecular Weight)	Dilution	Supplier
Spectrin alpha chain (nonerythroid) (AA6)	SPTAN1 (284 kDa)	1:1000	Sigma-Aldrich, Darmstadt, Hessen, Germany
phospho-p44/42 MAPK (Erk1/2) (197G2)	p-ERK (42/44 kDa)	1:1000	Cell Signaling Technology, Beverly, MA, USA
p44/42 MAPK (Erk 1/2) (137F5)	ERK (42/44 kDa)	1:1000	Cell Signaling Technology, Beverly, MA, USA
phospho-p38 MAPK (D3F9)	p-p38: (43 kDa)	1:1000	Cell Signaling Technology, Beverly, MA, USA
p38 MAPK (D13E1)	p38 (43 kDa)	1:1000	Cell Signaling Technology, Beverly, MA, USA
phospho-NF-κB p65 (93H1)	p-NFκB (65 kDa)	1:1000	Cell Signaling Technology, Beverly, MA, USA
NF-κB p65 (L8F6)	NFκB (65 kDa)	1:1000	Cell Signaling Technology, Beverly, MA, USA
VIM (polyclonal, HPA001762)	Vimentin (54 kDa)	1:1000	Sigma-Aldrich, Darmstadt, Hessen, Germany
E-Cadherin (M168)	E-Cadherin (135 kDa)	1:1000	Abcam, Cambridge, Cambridgeshire, UK
β-Aktin (AC-15)	β-Actin (42 kDa)	1:5000	Sigma-Aldrich, Darmstadt, Hessen, Germany

## Data Availability

The raw data supporting the conclusions of this article will be made available by the authors on request.

## Supplementary Materials

The following supporting information can be downloaded at https://www.mdpi.com/article/10.3390/ijms25115658/s1.
