# Supplementary material for: Erk Inhibition as a Promising Therapeutic Strategy for High IL-8-Secreting and Low SPTAN1-Expressing Colorectal Cancer
Source: Int J Mol Sci. 2024 May 23;25(11):5658. doi: 10.3390/ijms25115658 (PMC11172072; doi:10.3390/ijms25115658)
Supplement: Supplementary file 1 [file ijms-25-05658-s001.zip › ijms-2990030-supplementary.pdf]

Figure S1

A

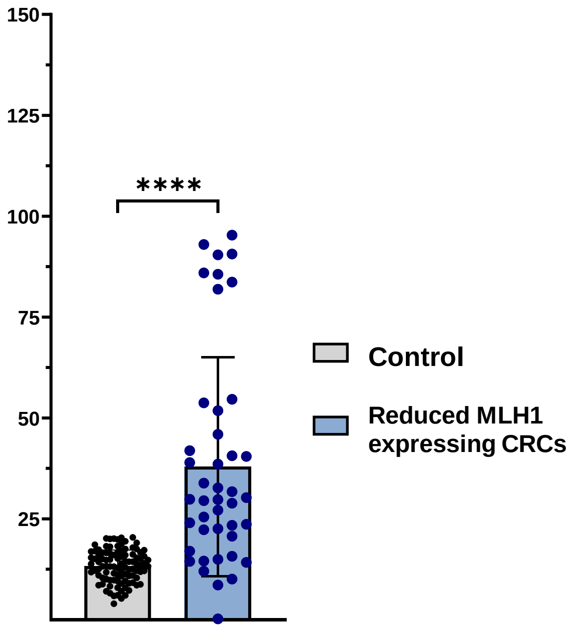

B

Table 1. CRC patients serum cytokine concentrations

CRC patients (n = 80)	IL-1β	IL-6	TNF-α
Median (pg/ml)	0,3775	4,454	0,779
25% Percentile	0,006445	2,093	0,4294
75% Percentile	0,6793	6,893	0,9838

Figure S2

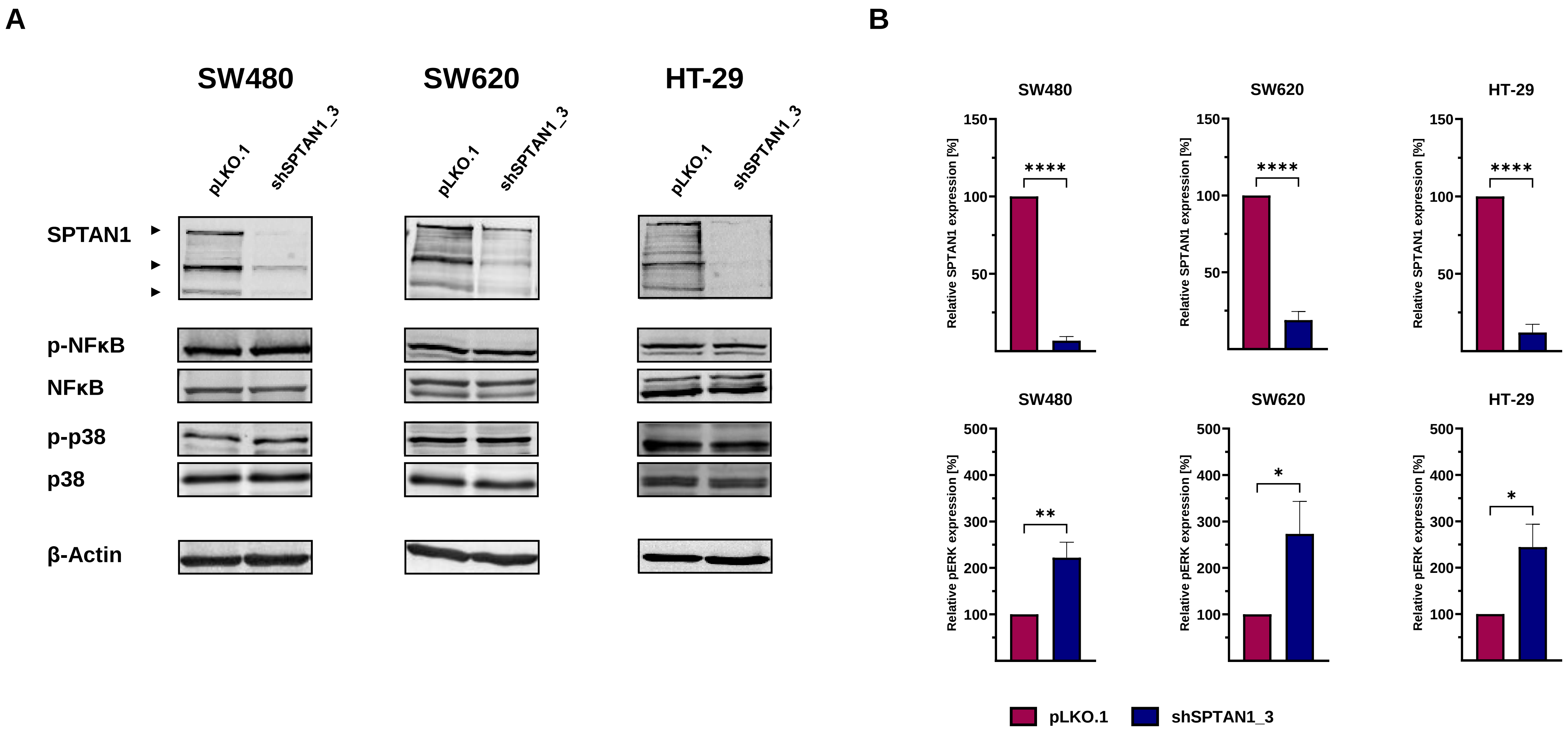
A
B
SW480
SW620
HT-29
pLKO.1
shSPTAN1_3
SPTAN1
p-NFκB
NFκB
p-p38
p38
β-Actin
Relative SPTAN1 expression [%]
Relative pERK expression [%]
150
100
50
500
400
300
200
****
**
*
pLKO.1
shSPTAN1_3

Figure S3

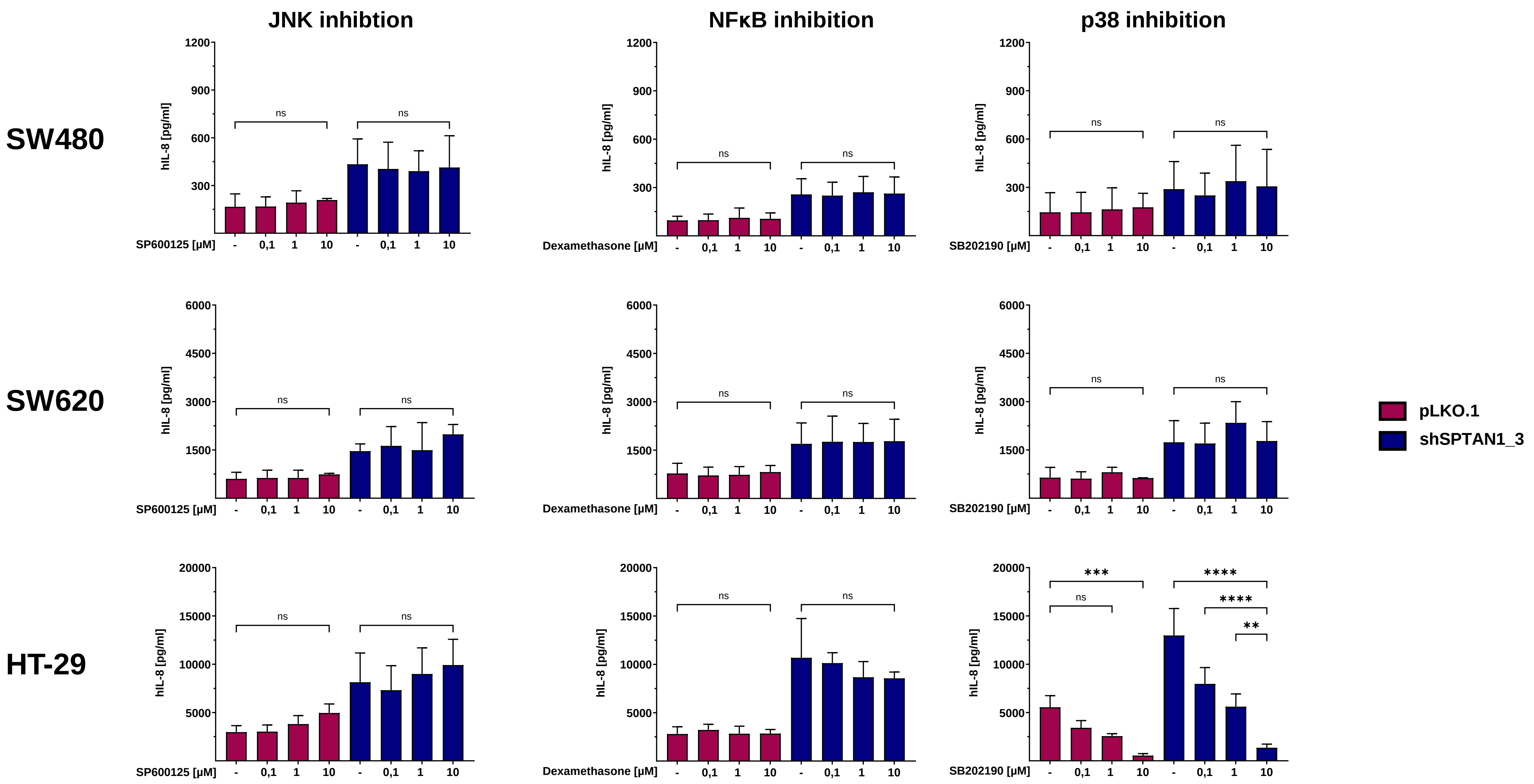

JNK inhibtion
NFκB inhibition
p38 inhibition
SW480
SW620
HT-29
hIL-8 [pg/ml]
SP600125 [µM]
Dexamethasone [µM]
SB202190 [µM]
- 0,1 1 10 - 0,1 1 10
ns
***
****
**
pLKO.1
shSPTAN1_3

Figure S4

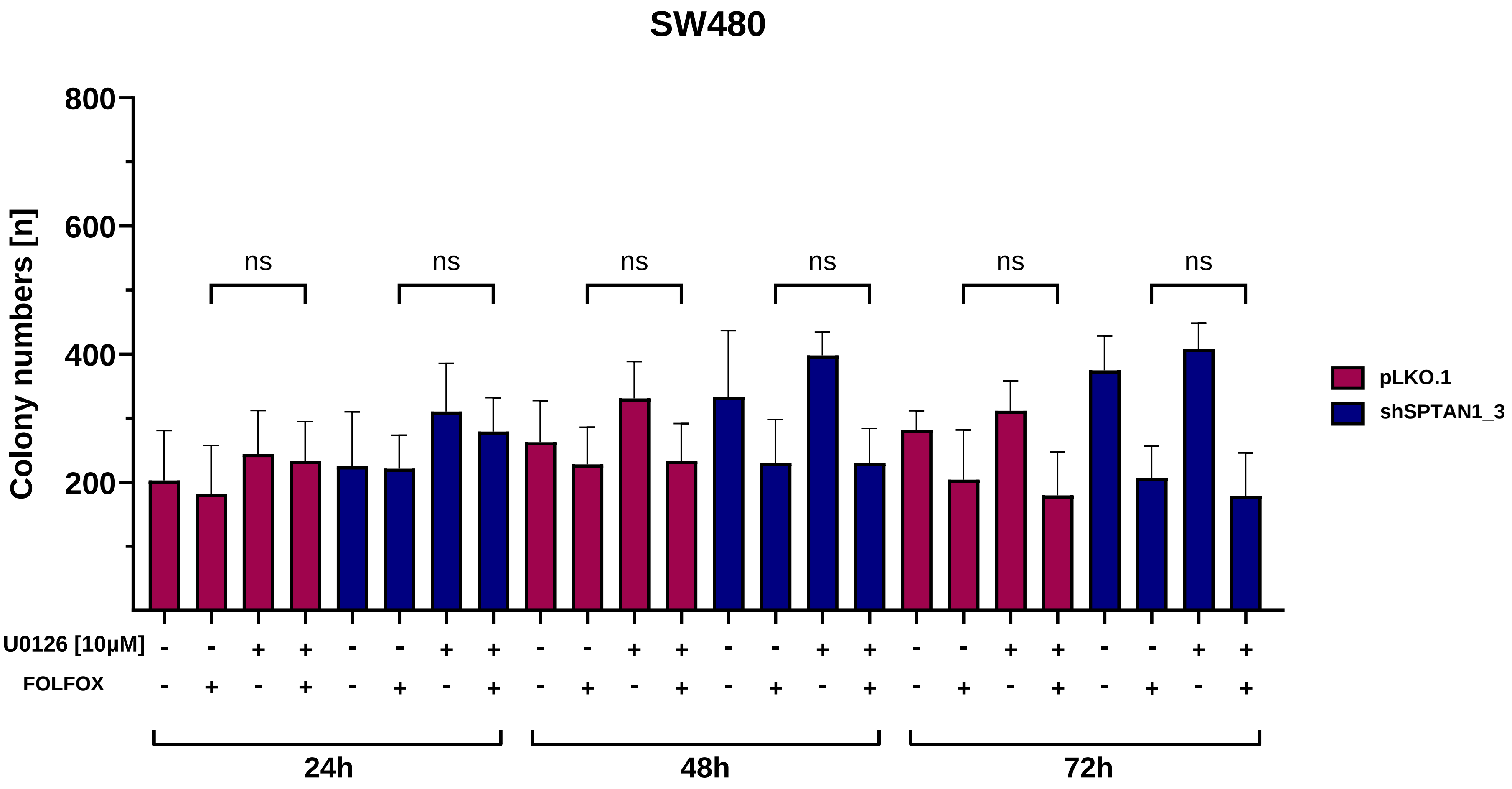
SW480
Colony numbers [n]
800
600
400
200
ns
ns
ns
ns
ns
ns
pLKO.1
shSPTAN1_3
U0126 [10µM]
- - + + - - + + - - + + - - + + - - + + - - + +
FOLFOX
- + - + - + - + - + - + - + - + - + - + - + - +
24h
48h
72h

Figure S5

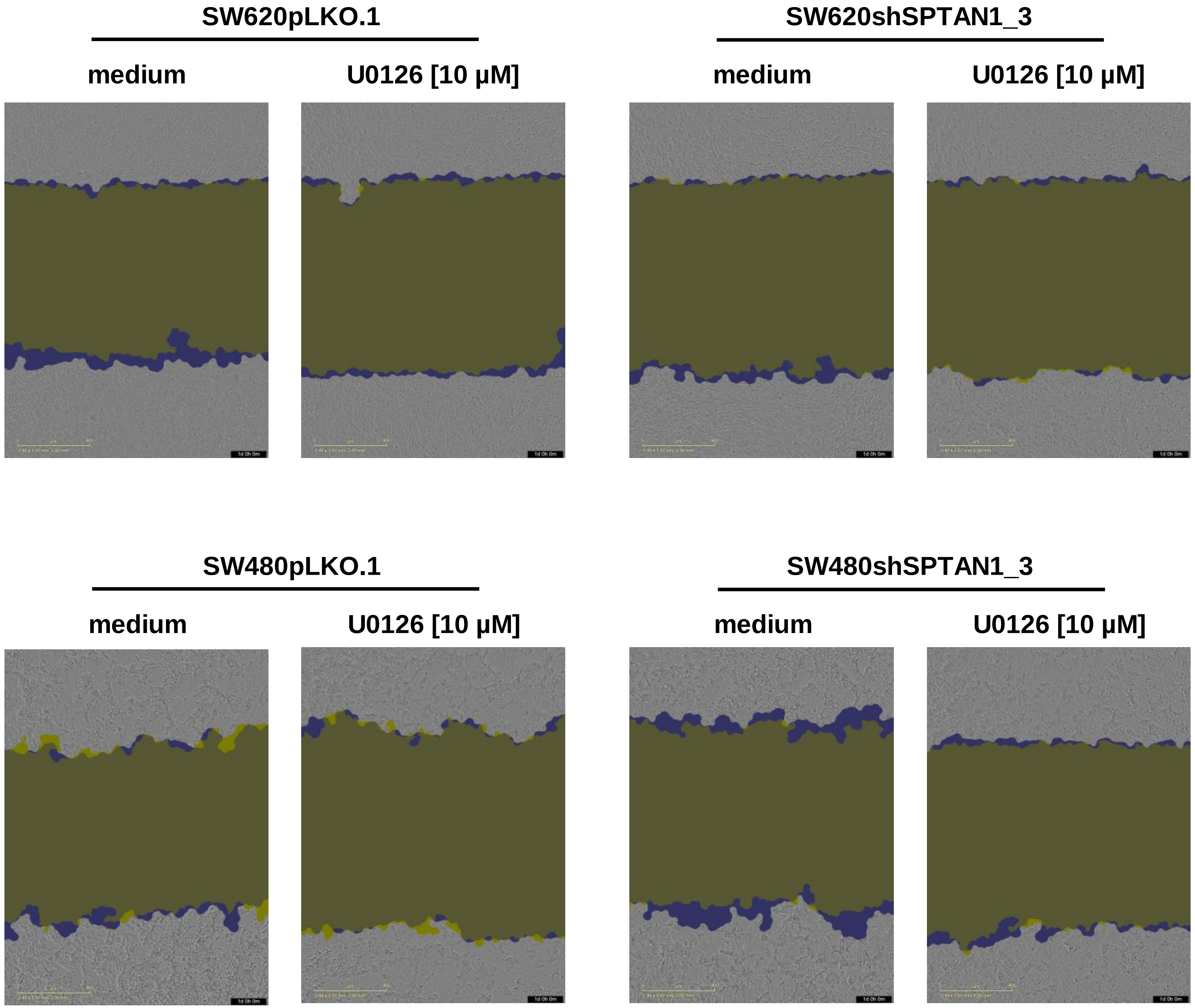
SW620pLKO.1
medium
U0126 [10 µM]
SW620shSPTAN1_3
medium
U0126 [10 µM]
SW480pLKO.1
medium
U0126 [10 µM]
SW480shSPTAN1_3
medium
U0126 [10 µM]

Figure S1: Reduced MLH1 expression is associated with significantly enhanced serum IL-8 concentrations in CRCs. Sera of 80 CRC patients were collected before tumor resection and compared with 100 control sera. IL-8 levels were determined by ELISA-measurements. After surgery, paraffin embedded CRCs (n = 75) of corresponding patients were analyzed for MLH1 expression via immunohistochemical staining. (A) IL-8 levels of reduced MLH1 expressing CRCs are significantly increased in comparison to the controls. (B) The levels of IL-1β, IL-6 and TNF-α were in the normal range and not elevated. P-values were calculated by Mann-Whitney-U test. **** p <0.0001

Figure S2. Significantly reduced SPTAN1 expression is associated with significantly increased ERK activation but not with the activation of NFκB or p38. Stable transduction of shRNA was used to reduce SPTAN1 expression in SW480, SW620 and HT-29 colon cancer cells, stable transduction of non-mammalian shRNA (pLKO.1) served as control, respectively. (A) Differential SPTAN1 expressing SW480, SW620 and HT-29 cell lines were cultivated, harvested and the expression of SPTAN1, NFκB, p-NFκB, p38 and p-38 was analyzed by Western blotting, exemplarily shown. No differences in the activation between reduced SPTAN1 expressing and pLKO.1 transfected cells could be detected. (B) The expression levels of SPTAN1, ERK and p-ERK of at least 3 independent Western blot experiments were quantified (mean ± SD) using Multi Gauge V3.2 program and normalized in relation to values of pLKO.1. shRNA transfected SW480, SW620 and HT-29 cells expressed significantly reduced SPTAN1 levels in comparison with the corresponding pLKO.1 transfected cells (for all $p < 0.0001$) and a significantly increased ERK phosphorylation could be detected in these reduced SPTAN1 expressing cells compared with the corresponding control cells (SW480 p = 0.0064; SW620 p = 0.0487, HT-29 p = 0.0265). P-values were calculated by unpaired T-test.

Figure S3. Increased IL-8 secretion of reduced SPTAN1 expressing cells is independent of JNK, NFκB and, with the exception of the HT-29, and p38. $5x10^5$ of differential SPTAN1 expressing SW480, SW620 and HT-29 cells were incubated for 48 h, then treated with the indicated concentrations of either JNK- (SP600125), NFκB- (Dexamethasone) or p38- (SB202190) specific inhibitor for 18 h, followed by ELISA-measurements. Columns show IL-8 concentrations of reduced SPTAN1 expressing cell lines (SW480 shSPTAN1_3; SW620 shSPTAN1_3; HT-29 shSPTAN1_3) (blue columns) in comparison to the pLKO.1 transduced controls (pink columns). Neither the inhibition of JNK or NFκB led to a reduction in IL-8 secretion. Only inhibition of p38 led to a significant reduction in IL-8 secretion in the HT-29 cell line. The data shown are means ± SD of at least three independent experiments. P-values were calculated by one-way ANOVA. The following p-values were considered as statistically significant: * $p < 0.05$, ** $p < 0.01$, *** $p < 0.001$.

Figure S4. FOLFOX and U0126 co-treatment slightly reduces long-term survival of reduced SPTAN1 expressing cells. 1×10^3 SW480 cells were seeded per cell culture flask (surface area 25 cm^2), incubated until adhesion and cultivated in medium supplemented with or without 10 µM of U0126 for 18 h. Subsequently, cells were treated with FOLFOX supplemented with or without 10 µM of U0126 for 24 h, 48 h and 72 h. The medium was replaced and cells were cultivated in medium without supplements for further seven days. Colonies were fixated with 4% paraformaldehyde, and stained with 0.5% crystal violet. The number of colonies was quantified using the COLCOUNT™ system and the Oxford Optronix ColCount software version 4.3.5.1. Columns show the number of colonies of reduced SPTAN1 expressing cell lines (blue columns) in comparison to the pLKO.1 transduced controls (pink columns). Co-treatment of U0126 and FOLFOX slightly reduces the colony formation of all cell lines after 72 h. P-values were calculated by one-way ANOVA; n = 3. The data shown are means ± SD, the following p-values were considered as statistically significant: ns = not significant

Figure S5. Inhibition of ERK impairs cell migration. A wound migration assay was used to test the efficacy of ERK inhibition by 10 µM U0126 treatment on cell mobility of differential SPTAN1 expressing cells. Exemplary images of the cell migration of reduced SPTAN1 expressing cells (SW480

shSPTAN1_3 and SW620 shSPTAN1_3, right side) as well as corresponding pLKO.1 transduced control cells (left side) treated with or without 10 μM U0126 are presented. The purple line indicates the area of the initial scratch and the yellow line shows the scratch after 24 hours. The difference indicates the migration of the cells.

Table S1. Clinical Features and IL-8 serum Levels in 80 Primary Colorectal Carcinomas

**Case**	**Age (years)**	**Sex**	**IL-8 (pg/ml)**	**MLH1'**	**SPTAN1''**	**PD-L1'''**
IL-8 001	74	F	90.63	-04.09	-09.39	24.37
IL-8 003	85	F	33.88	-31.92	-31.21	41.41
IL-8 004	81	F	40.65	-28.65	14.36	54.34
IL-8 005	68	M	08.57	89.18	00.23	01.96
IL-8 006	75	M	19.73	11.97	47.18	-04.58
IL-8 007	83	F	39.43	20.72	-24.18	03.00
IL-8 008	55	F	15.33	50.72	19.33	-05.88
IL-8 009	85	F	90.44	-14.98	12.34	07.18
IL-8 010	44	F	08.64	-01.25	08.69	-09.18
IL-8 011	84	M	24.05	-02.36	15.22	05.63
IL-8 012	85	F	54.63	-63.13	-58.30	69.89
IL-8 013	89	M	23.65	-16.08	10.19	24.43
IL-8 014	63	M	25.49	-63.74	-33.44	63.09
IL-8 015	84	F	85.96	-80.56	-43.41	
IL-8 016	67	M	16.62	07.33	14.32	02.72
IL-8 018	85	F	14.49	-04.44	23.89	-05.05
IL-8 019	70	M	53.74	-34.93	-16.68	-04.83
IL-8 020	66	F	83.67	-21.85	-14.88	00.34
IL-8 021	54	M	31.76	-10.90	-34.91	-03.65
IL-8 022	65	M	29.73	13.98	-12.35	04.91
IL-8 024	72	F	22.68	33.56	02.47	-08.28
IL-8 025	72	F	12.02	-20.62	32.62	08.16
IL-8 026	77	M	20.45	18.56	09.15	03.69
IL-8 027	58	M	14.02	20.82	04.38	22.67
IL-8 028	78	F	11.03	15.81	12.15	09.55
IL-8 029	60	F	20.78	02.04	03.65	-01.91
IL-8 030	54	M	51.31	01.70	37.39	17.85
IL-8 031	60	M	28.89	-03.99	-22.92	05.41
IL-8 032	61	M	51.82	-12.48	-20.25	05.78
IL-8 033	80	M	00.06	39.90	47.51	-08.70
IL-8 034	65	F	00.24	-12.86	04.98	-01.36
IL-8 035	67	F	06.36	31.54	05.24	19.76
IL-8 037	56	M	23.63	27.65	00.18	08.20
IL-8 038	82	M	25.20	19.48		01.80
IL-8 039	61	M	85.61	-50.18	19.73	-02.42
IL-8 040	85	M	17.00	-21.26	28.35	-06.15
IL-8 041	60	F	00.00	-27.99	22.57	01.18
IL-8 043	72	M	16.52	06.79	02.18	-01.29
IL-8 044	71	F	38.58	-10.48	20.64	-01.79
IL-8 045	63	M	29.69			
IL-8 046	74	F	45.97	-18.89	-00.26	-00.97
IL-8 047	54	F	81.93	-23.72	-60.47	01.09
IL-8 053	82	M	10.91	06.11	28.26	13.59
IL-8 055	64	F	40.50	-18.45	35.47	23.83
IL-8 057	77	F	41.92	-16.02	48.51	07.24
IL-8 059	51	M	22.31	-0.77	02.94	01.13
IL-8 062	93	F	40.46	47.49	03.86	03.47
IL-8 063	82	M	24.75	10.57	09.22	05.06

IL-8 064	68	F	08.12	35.83	36.07	02.41
IL-8 065	75	F	15.73	-00.08	02.56	-01.72
IL-8 066	79	F	22.58	-42.88	09.02	-03.15
IL-8 067	65	F	29.54	-40.27	-42.21	-01.18
IL-8 068	64	F	26.18	08.08	50.64	02.80
IL-8 070	75	F	24.38	10.59	-13.88	00.93
IL-8 072	49	F	27.17	-17.66	-4.21	-02.87
IL-8 073	72	F	61.51	08.04	-22.59	-02.04
IL-8 074	58	M	20.70	-39.67	21.61	-03.03
IL-8 075	75	M	22.67	18.54	33.10	00.68
IL-8 076	79	F	30.28	-07.76	31.61	-02.89
IL-8 078	57	F	12.74	00.97	12.45	-01.30
IL-8 079	70	M	11.94			
IL-8 080	61	F	20.77	18.44	03.18	00.95
IL-8 081	81	M	08.03			
IL-8 082	79	F	64.73			
IL-8 083	68	M	23.87	27.81	60,29	00.01
IL-8 084	76	F	10.11	-07.43	00.41	-00.62
IL-8 085	76	M	23.41	-45.00	07.73	03.31
IL-8 086	58	M	17.10	00.45	19.09	09.97
IL-8 088	82	M	40.61	22.34	16.10	01.46
IL-8 089	63	M	14.22	-02.27	08.23	04.86
IL-8 090	62	M	32.69	-32.59	12.97	-03.52
IL-8 092	67	M	29.85	-63.02	-61.53	01.41
IL-8 093	52	M	95.33	-27.05	-50.72	22.85
IL-8 094	86	F	38.96	-16.85	-42.08	-03.19
IL-8 095	57	F	08.79	32.58	51.72	03.91
IL-8 096	51	M	14.97	-01.58	11.52	-03.06
IL-8 097	85	F	14.58	-53.62	-16.46	63.96
IL-8 098	85	M	92.98	-18.82	-29.60	22.70
IL-8 099	58	F	29.78	-20.50	18.58	-01.15
IL-8 102	64	M	12.62	40.76	15.55	10.49

F, female; M, male

‘; ‘‘; ‘‘‘ Difference between tumor and tumor surrounding normal mucosa
